# Novel Alkyl-Polyglucoside-Based Topical Creams Containing Basil Essential Oil (*Ocimum basilicum* L. Lamiaceae): Assessment of Physical, Mechanical, and Sensory Characteristics

**DOI:** 10.3390/pharmaceutics17070934

**Published:** 2025-07-19

**Authors:** Ana Barjaktarević, Georgeta Coneac, Snežana Cupara, Olivera Kostić, Marina Kostić, Ioana Olariu, Vicenţiu Vlaia, Ana-Maria Cotan, Ştefania Neamu, Lavinia Vlaia

**Affiliations:** 1 Department of Pharmacy, Faculty of Medical Sciences, University of Kragujevac, 34000 Kragujevac, Serbia; ana.radovanovic@medf.kg.ac.rs (A.B.); cupara@fmn.kg.ac.rs (S.C.); olivera.kostic@fmn.kg.ac.rs (O.K.); 2 Department II—Pharmaceutical Technology, Formulation and Technology of Drugs Research Center, Faculty of Pharmacy, “Victor Babeș” University of Medicine and Pharmacy, 300041 Timișoara, Romania; coneac.georgeta@umft.ro (G.C.); mut.anamaria@umft.ro (A.-M.C.); adriana.colgiu@umft.ro (Ş.N.); vlaia.lavinia@umft.ro (L.V.); 3 Center for Harm Reduction of Biological and Chemical Hazards, Faculty of Medical Sciences, University of Kragujevac, 34000 Kragujevac, Serbia; marrina2006kg@yahoo.com; 4 Department of Pharmacology and Toxicology, Faculty of Medical Sciences, University of Kragujevac, 34000 Kragujevac, Serbia; 5 Department II—Organic Chemistry, Formulation and Technology of Drugs Research Center, Faculty of Pharmacy, “Victor Babeș” University of Medicine and Pharmacy, 300041 Timișoara, Romania; 6 Pharmacy Doctoral School, Faculty of Pharmacy, “Victor Babes” University of Medicine and Pharmacy, 300041 Timisoara, Romania

**Keywords:** basil essential oil, alkyl polyglucoside, creams, physicochemical, mechanical, sensory properties

## Abstract

**Background/Objectives:** Basil essential oil exhibits a wide range of biological activities, including strong antimicrobial and anti-inflammatory effects. Considering the health benefits of basil essential oil (BEO) and the favorable properties of alkyl polyglucoside emulsifiers, novel Montanov™-68-based O/W creams containing BEO were developed and characterized. Additionally, the influence of the emulsifier content on the cream’s properties was evaluated. **Methods**: The physicochemical properties were evaluated by organoleptic examination, physical stability test, and pH and electrical conductivity measurement. The mechanical properties were investigated by rheological, textural, and consistency analyses. In addition, a sensory evaluation protocol was applied. **Results**: The cream formulations containing 5% and 7% Montanov™ 68 demonstrated physical stability, with no evidence of phase separation during the observation period or following accelerated aging. The pH values remained within the acceptable range for topical use, and a gradual decrease in electrical conductivity over time was observed. The rheological analyses confirmed the non-Newtonian pseudoplastic behavior with thixotropic flow characteristics. The textural analyses demonstrated that the higher emulsifier content led to increased firmness, consistency, cohesiveness, and index of viscosity. The sensory analysis revealed differences between the alkyl polyglucoside (APG)-based cream formulations only in terms of the elasticity and stickiness. **Conclusions**: Although the rheological analyses suggested the better spreadability of the formulation with 5% emulsifier, this was not confirmed by the sensory analysis. However, the APG-based formulations performed significantly better than the synthetic surfactant-based formulation in terms of the absorption, stickiness, and greasiness (during and after application). These results are encouraging for the further evaluation of APG-based creams containing basil essential oil for topical application.

## 1. Introduction

Natural products have been used for centuries in the treatment and prevention of various diseases due to their health benefits [1]. According to the World Health Organization, more than 80% of the global population currently relies on some form of traditional medicine [2]. In support of this, plant essential oils are being studied as a promising alternative to synthetic drugs, particularly given the growing concerns over drug resistance and potential side effects [3]. *Ocimum basilicum*, commonly known as sweet basil, is an aromatic herb belonging to the genus Ocimum and the Lamiaceae family. It is native to regions of Asia, Africa, and South America [4]. It is extensively reported that *O. basilicum* essential oil (BEO) exhibits numerous biological activities, including antioxidant, antimicrobial, antiviral, anti-inflammatory, and analgesic effects [1,5]. However, the antimicrobial effect of BEO is the most thoroughly investigated, showing antibacterial activity against both Gram-positive and Gram-negative bacteria, antifungal activity, as well as multi-resistant clinical isolates of certain bacterial strains [6,7,8]. In addition, the significant anti-inflammatory and antiedematogenic effects of BEO have been confirmed in an in vivo animal model of paw edema [9]. Various carriers, including hydrogel, emulgel, and bioactive sponge, have been developed with BEO as an active ingredient, which when topically applied, demonstrate wound-healing activity in animal wound models [7,10]. These biological activities are largely attributed to the presence of bioactive compounds in the BEO, particularly phenylpropanoid compounds (eugenol, methyl eugenol, estragole, and methyl cinnamate) and terpenoids (oxygenated sesquiterpenes, oxygenated monoterpenes, sesquiterpene hydrocarbons, monoterpene hydrocarbons) [5,8]. The chemical composition varies depending on the *O. basilicum* chemotypes. The most represented are chemotypes of BEO, primarily composed of estragole, and the one consisting mainly of linalool and estragole [6]. Although the daily intake of BEO is associated with a potential risk to human health due to its high content of alkenylbenzenes, topical use is considered safe [11]. Stanojevic et al. showed that basil essential oil was not toxic to human cells and did not cause irritation in a skin irritation model [11,12].

To the best of our knowledge, semisolid topical products containing BEO have been developed as stearic-acid-based cream, macrogol-based ointment, emulgel, and hydrogel [7,13]. The literature provides no data on an alkyl-polyglucoside-based cream containing BEO. Alkyl polyglucosides (APGs) are an FDA-certified class of non-ionic, oil-in-water (O/W), biodegradable emulsifiers derived from natural, renewable sources. The dermatological benefits of APG emulsifiers, including biocompatibility, enhanced skin barrier function, prolonged moisturizing effect, no erythematous potential, and environmentally friendly profile, support the preference for APG emulsifiers in compounding over conventional surfactants [14,15]. An alkyl polyglucoside emulsifier with the commercial name Montanov™ 68 was used to formulate the O/W cream containing BEO. According to the manufacturer (Seppic, France), Montanov™ 68 (INCI name: cetearyl alcohol and cetearyl glucoside) is a naturally derived emulsifier sourced from palm or coconut oil and corn [16]. A particular advantageous property of this emulsifier is its ability to form lamellar liquid crystal emulsions, which in comparison to classic emulsions, present improved physical stability, rheological and moisturizing properties, and a better skin sensory feeling [17]. This novel type of emulsion contains lamellar liquid crystals formed by some emulsifiers, which arrange themselves in ordered bilayers around the oil droplets at the oil–water interface; the lamellar bilayers act as an effective barrier against coalescence. The liquid crystals can also be found in the aqueous phase as lamellar phases, viscoelastic and more or less consistent networks, which hinder the creaming phenomenon [18].

Considering the health benefits of basil essential oil and the favorable properties of APG emulsifiers, an alkyl-polyglucoside-based cream containing BEO could be of interest for future research as a promising topical product for the prevention, treatment, or adjunctive therapy of various skin conditions. Therefore, this study introduces a novel, biocompatible, and environmentally sustainable emulsifier system as an alternative to the conventional anionic and non-ionic surfactants traditionally used in topical formulations but potentially associated with skin safety concerns.

Considering the previously provided data, our research aimed to develop a novel Montanov™-68-based O/W cream containing basil essential oil with two different surfactant concentrations. As a first step in this research, the present study’s objective was to assess the physicochemical, mechanical, and sensory properties of the developed formulations, along with the influence of the emulsifier content on these properties.

## 2. Materials and Methods

### 2.1. Materials

According to the manufacturer, the basil essential oil (Essenciagua, La Tieule, France) is obtained by distillation of the aerial parts of *O. basilicum* L. The Montanov™ 68 was kindly donated by Seppic, Paris, France. The oil phase components, including caprylic/capric triglyceride, cetyl palmitate and almond oil, as well as xanthan gum, were purchased from AvenaLab Cosmetics, Vršac, Serbia. The components of the aqueous phase were sodium sorbate, propylene glycol (Häffner GmbH & Co., Asperg, Germany), and purified water. The tocopheryl acetate, polysorbate 60, cetylstearyl alcohol, glycerol, and Vaseline were also purchased from AvenaLab Cosmetics, Vršac, Serbia.

### 2.2. Methods

#### 2.2.1. Formulation of APG-Based Emulsions

The investigated cream formulations were oil-in-water (O/W) emulsions. The preparation method involved separately preparing the oil and water phases. The APG emulsifier (Montanov™ 68), along with the oil phase components, was heated to 75 °C (magnetic stirrer IKAMAG, IKA-Werke GmbH & Co., Staufen, Germany). The xanthan gum was added to the water phase, which was heated at 2–5 °C higher than the oily phase, and stirred with a propeller stirrer until dissolved. Emulsification was achieved by gradually adding the water phase to the oil phase while stirring continuously with a propeller mixer. The mixture was stirred for 2 min at 4000 rpm, followed by 2 min at 800 rpm, and then at 500 rpm until cooled. The tocopheryl acetate and BEO were added after the cream cooled below 40 °C. The reference non-ionic hydrophilic base was prepared according to DAB 2006 [19]. The control sample was composed of a reference non-ionic hydrophilic base, water phase, preservative, and basil essential oil. It was intended for comparison of the sensory properties with the sample formulations, and it was not subjected to the physical and mechanical characterization. The composition of all the cream formulations is presented in Table 1. The analyses were performed 7 days after the preparation of the APG-based cream samples, when equilibrium was achieved.

#### 2.2.2. Physicochemical Properties of Cream Formulations

Through visual inspection, the APG-based formulations containing BEO were characterized organoleptically, including the appearance, color, odor, gloss, homogeneity, and instability signs such as creaming and phase separation. The creams were packaged into plastic containers and stored at room temperature. The examination was conducted at 7 and 30 days after preparation. In addition, visual control was performed during all the phases of the accelerated aging test.

The physical stability was analyzed using a centrifuge assay, performed in two cycles at 3000 rpm for 15 min (Hettich Mikro 120, Andreas Hettich GmbH, Tuttlingen, Germany), with phase separation checking. The assessment was repeated at 7, 30, and 90 days after production, and following the accelerated aging test [20].

The pH values of the experimental creams were measured in triplicate at 25 ± 2°C, using a calibrated SevenExcellence™ S400-KIT pH meter (Mettler Toledo, Columbus, OH, USA) at 7, 30, and 90 days after production. The official potentiometric procedure was applied [21]: 1 g of each cream was dispersed by stirring for 15 min in 20 mL of purified water; afterward, the obtained dispersion was filtered, and the pH of the filtrate was determined.

The electrical conductivity was measured according to the European Pharmacopoeia (2.2.38) official method with some modifications [22]. The test was performed using the calibrated conductometer (Eutech CON 700, Thermo Fisher Scientific, Shanghai, China), suitable for semisolid products, by direct immersion of the electrode into the experimental creams at room temperature (20 ± 2 °C), after 7, 30, and 90 days.

The accelerated aging test consisted of three cycles of 24 h, with the experimental creams exposed to room temperature, followed by 5 ± 2 °C, and 45 ± 2 °C. After the final cycle, the samples underwent visual inspection and pH and electrical conductivity measurements [20].

#### 2.2.3. Mechanical Properties of Cream Formulations

##### Rheological Analysis

The rheological properties of the experimental creams were studied by both steady shear experiments and dynamic oscillatory experiments, using a stress-controlled rheometer (RheoStress 1, Thermo Haake, Karlsruhe, Germany) equipped with a cone-plate geometry (C35/2Ti, 35 mm diameter, 2 cone angle and 0.105 mm gap size) and a Peltier module (TCP/P) for temperature control (23 °C). Analysis of the rheological data was performed using HAAKE RheoWin Data Manager 4 version 4.3.

##### Steady Shear Tests

In the steady shear experiments, the shear stress (τ, in Pa) and the apparent viscosity (η, in Pa·s) were measured as a function of the shear rate ($\dot{\gamma }$ in 1/s). The measurements were conducted in controlled rate mode with a continuous ramp up and down by progressively increasing the share rate from 0.05 1/s to 100 1/s during 120 s, holding the maximum value for 10 s, and then decreasing from the maximum to the minimum value of this range in another 120 s. Thereby, the area under the upward flow curve (A_UP_) and the area below the downward flow curve (A_Down_) were calculated. By means of regression analysis of the flow data using the Ostwald de Waele and Herschel–Bulkley rheological models (Equations (1) and (2)), the consistency (K) and flow (n) indices were calculated; the model with the higher regression coefficient (R^2^) was selected.

(1) $$\tau =K\;\cdot {\dot{\gamma }}^{n}$$

(2) $$\tau =\tau_{0}+K\cdot {\dot{\gamma }}^{n}$$

Using the values of A_UP_ and A_Down_, the thixotropy index (relative area of hysteresis, A_R_, %), quantifying the thixotropy of the tested creams, was calculated using the following Equation (3):

(3) $$A_{R}=\frac{\left(A_{UP}-A_{Down}\right)}{A_{UP}}\cdot 100$$

It is considered that systems are thixotropic if the A_R_ values are higher than 5% and are only pseudoplastic with no thixotropy if the A_R_ values are less than 5% [23].

##### Dynamic Oscillatory Tests

The cream samples were tested in terms of the viscoelasticity, undergoing the following tests: the oscillatory stress sweep test and the oscillatory frequency sweep test. First, the oscillatory stress sweep test was conducted at a frequency of 1.59 Hz, at different shear stress domains (0.5–10 Pa, 0.5–100 Pa, 0.5–500 Pa), to assess the linear viscoelasticity region (LVR) of the samples by measuring the shear moduli (storage modulus G’ and loss modulus G″) as a function of the shear stress, τ. Subsequently, an oscillatory frequency sweep test was performed at a constant shear stress of 5.0 Pa (selected from the LVR region), over a wide frequency domain (0.05–50 Hz), and the G’ and G″ moduli and the complex viscosity (η*) were determined [24]. To determine the yield stress, new untested cream samples were used and the oscillatory stress sweep test was carried out at a constant frequency of 1 Hz and by increasing the shear stress from 1 Pa to 500 Pa, which allowed the acquisition of the G’ and G” profiles as functions of the shear stress, τ, and consequently, the measurement of the yield stress (τ0) of the experimental creams. The yield stress is the shear stress value at which a sharp drop in G’ is observed, indicating that an irreversible plastic deformation has occurred [25]. Each of the rheological tests (steady shear and dynamic oscillatory experiments) was performed in triplicate.

##### Texture Analysis of Experimental Creams

A texture analyzer equipped with a 5 kg load cell (TAXT Plus, Stable Micro Systems, London, UK) was used to assess several textural properties of the cream formulations by two tests.

For the back extrusion compression test, an extrusion probe (a compression disc with a diameter of 35 mm, attached to a rod) was used, and all the experiments were performed at room temperature and in compression mode. The steps of the test protocol were as follows: a specific amount (50 g) of cream sample was weighted in a 100 mL container, reaching a height of 50 mm, without air incorporation; the extrusion probe was lowered to the surface of the sample and a trigger force (5 g) was applied to detect contact with the sample; the probe continued to descend in the sample at a speed of 2 mm/s, to a depth of 15 mm; after reaching the indicated test depth, the probe returned to the cream surface with the same speed (2 mm/s). Data recording and construction of the force–time profiles were performed using the software Texture EXPONENT version 3.0.5.0. The force–time curve permitted the calculation of the following texture parameters [26]: firmness (in g) defined as the maximum force required for sample deformation and indicated by the maximum value of the compression depth of the probe; consistency (in g·s), defined as the area of the positive region referring to the total work required for compression; cohesiveness (in g), defined as the maximum force required to withdraw the probe from the sample to the surface; and viscosity index (in g·s), defined as the area of the negative region.

A specific accessory (TTC Spreadability Rig HDP/SR, Stable Micro Systems, London, UK) was used for the spreadability test. The work procedure included the following steps: the male-type conical probe was lowered at a speed of 3 mm/s at 2.3 cm from the surface of the sample, placed in the female-type conical support; afterward, the male-type cone penetrated the sample, pressing out the cream at 45° between the surfaces of the two cones (male and female). The instrument software measured the force (g) required for the male-type conical probe to penetrate the sample. The plot of the force values versus the time provided two important parameters indicating the firmness and spreading capacity of the creams: the maximum force value and the shear work corresponding to the area under the curve in the positive region, respectively. Low force and work of shear values correspond to better spreadability. For both texture analyses, four samples of each cream formulation were tested at ambient temperature, and the results are shown as the average ± standard deviation (SD).

##### Consistency Tests

For supplementary evaluation of the consistency properties of the experimental creams, two specific tests were performed: penetrometric test (compendial method) [27] and parallel-plate method.

The penetrometric test was conducted to evaluate the hardness, a component of the structural strength of semisolid products, using a penetrometer (PNR 12, Petrolab, Speyer, Germany) equipped with a penetrating microcone and a suitable container. The assay was carried out at 25 ± 2 °C, according to the procedure and conditions described in the pharmacopeia; the penetration depth is expressed in millimeters (mm), as the arithmetic mean of the 3 measurements ± SD. The hardness of a semisolid is inversely proportional to the penetration depth of the cone.

The parallel-plate method was used to determine the spreading ability of the experimental creams, using the del Pozo Ojeda-SuñéArbussá extensometer, following the procedures described in the literature [28]. The results are presented as spreading profiles by plotting the surface areas (mean ± SD) produced by the cream samples as a function of the applied standardized weights. All the measurements were performed at 25 ± 2 °C, in triplicate.

#### 2.2.4. Sensory Properties of Cream Formulations

This study of the evaluation of the sensory properties of the cream formulations was approved by the Ethics Committee of the Faculty of Medical Sciences, University of Kragujevac, Serbia, No. 01-1743/4 from 6 March 2024. The examination was conducted in a laboratory with proper lighting, at a room temperature of 21 ± 2 °C, and at a relative humidity of 45 ± 3%. The sensory characteristics of the cream formulations were evaluated by 20 participants (aged between 20 and 30 years). The inclusion criteria were personal interest in participation and the ability to verbally describe the feeling with descriptive terms. For each sensory attribute, a determination procedure was presented using materials that represent the extremes of certain sensory attributes. Then, the assessment of the sensory attributes of the samples was carried out by the panelists, who completed a questionnaire consisting of three sets of questions, each addressing questions regarding the samples before, during, and after the application to the skin. The assessors responded using defined descriptive terms or a scale ranging from 1 to 10 (Table 2) [29,30,31]. All the samples were available in identical plastic containers, and blind examination was conducted.

#### 2.2.5. Statistical Analysis

The statistical analyses were performed using IBM^®^ SPSS Statistics version 21.0. The Mann–Whitney test and the Wilcoxon signed-rank test for paired samples were used for the statistical analyses where applicable. For the analysis of the sensory characteristics of the formulations, the Chi-square test, the Friedman test, and the sum of ranks with multiple sample comparisons using Fischer’s LSD test were applied. High rank sums correspond to a high level of perception for each sensory attribute. Differences were considered statistically significant if *p* < 0.05 and highly significant if *p* < 0.01.

## 3. Results

### 3.1. Physical Properties of Cream Formulations

By visual observation, the formulations (F_1_ and F_2_) were characterized as homogeneous, semisolid in consistency, white in color, and pearlescent in appearance, with the presence of a characteristic odor derived from the essential oil of *Ocimum basilicum* L. Formulation F_1_, containing a lower emulsifier concentration (5%), was relatively thick, whereas sample F_2_, with a higher emulsifier concentration (7%), exhibited a heavier and thicker consistency.

The physical stability of all the examined samples was evaluated using the centrifugation method at 7, 30, and 90 days post-preparation, and after an accelerated aging test. No phase separation was observed in the samples, indicating good physical stability.

The pH value and electrical conductivity of the samples, measured at 7, 30, and 90 days after production, as well as after the accelerated aging stability test, are presented in Table 3. Throughout the 3-month observation period, a slight decrease in the pH values was observed. The changes in the pH value during the time of observation were not significant (*p* > 0.05).

Although it is common for oil-in-water emulsions to have electrical conductivity values above 50 µS/cm, our samples showed much lower values (Table 3). For different oil-in-water emulsion formulations, several previously published studies reported electrical conductivity values around or higher than 50 µS/cm, which were attributed to their composition: water and oil concentration, type and concentration of surfactant emulsifiers, and other ingredients [32,33,34]. Also, it is well known that when the conductivity values are higher than 10 µS/cm, the emulsion has water as the continuous phase, whereas conductivity values lower than 10 µS/cm indicate that the continuous phase of the emulsion is oil [35,36]. Correlating our results with the abovementioned observations, it can be noted that the F_1_ and F_2_ cream formulations are oil-in-water emulsions, with electrical conductivity values two to three units higher than 10 µS/cm. The small differences between the conductivity values of the creams can be attributed to the slight increase in the surfactant’s concentration. Both samples exhibited a decrease in electrical conductivity over the observation period, with the values recorded after 90 days closely resembling those obtained following the accelerated aging test. At the end of the observation period, the electrical conductivity values remained below 50 µS/cm, consistent with those expected for emulsions featuring lamellar liquid crystals.

### 3.2. Rheological Analysis

#### 3.2.1. Steady Shear Tests

**Figure 1:**
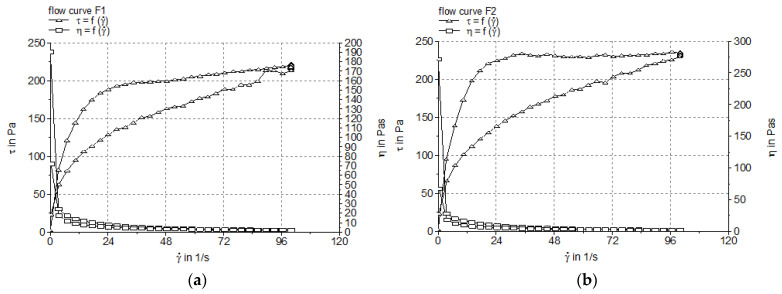
Flow and viscosity profiles of the APG-based creams containing basil essential oil: (**a**) formulation F_1_—Montanov 68 5% + BEO 1%; and (**b**) formulation F_2_—Montanov 68 7% + BEO 1%.

The rheograms and viscosity profiles of the experimental creams based on the APG emulsifier (Figure 1) indicated the complex rheological behavior of these systems, namely non-Newtonian, pseudoplastic (shear-thinning), and thixotropic. The shear stress showed a linear dependence on the deformation rate, and the viscosity decreased with the increase in the deformation rate. In addition, the presence of the hysteresis loop in the rheograms suggests that the viscosity decreased gradually when shear stress was applied and increased in time after shearing was stopped, recovering its initial state after a resting time [37].

The viscosity and thixotropy index values of the experimental creams are summarized in Table 4, while Table 5 lists the mean values of the rheological parameters obtained by fitting the viscosimetric test results with two rheological models, namely Ostwald de Waele and Herschel–Bulkley (the fitting was performed for the upward flow curve A_UP_).

The apparent viscosity values for the cream samples (Table 4) were measured at the maximum shear rate (100 1/s). Generally, a shear rate of 100 1/s is considered the reference point for the assessment of the flow behavior of non-Newtonian systems, for which the apparent viscosity varies with the shear rate. It is considered that the measurement of the apparent viscosity at 100 1/s offers a better understanding of the flow behavior of different non-Newtonian materials in real-world applications (i.e., in the conditions operating during the application of ointments, creams or gels to the skin) [38,39]. The continuous shear measurements revealed that both APG-based creams with BEO exhibited pronounced thixotropy (Figure 1, Table 4). This rheological property, correlated with the material microstructure, is desirable for topical semisolid preparations, as it reveals that during application on the skin (under the action of a shear stress), the semisolid product becomes thinner and can be easily spread. An increase in the Montanov 68 surfactant concentration from 5% to 7% corresponded to a very slight rise in the thixotropy index, indicating the physical stability of the microstructure. Based on the results presented in Table 5, the Herschel–Bulkley model described more accurately than the Ostwald de Waele model the flow properties of the investigated creams, as it resulted in higher R^2^ values. The values of the flow index (n) for this rheological model revealed the pseudoplastic behavior of the samples, considering that 0 < n <1. As is known, the lower the n values, the more pseudoplastic the system. Therefore, one can suggest that the cream formulation with a higher concentration of Montanov 68^®^ emulsifier exhibited a pronounced shear-thinning behavior, as indicated by the lower n value compared to the formulation with 5% Montanov™ 68 surfactant (0.1433 ± 0.005 and 0.1948 ± 0.011, respectively) (Table 5). However, for the cream formulations containing the Montanov 68™ emulsifier, the consistency index calculated with the Herschel–Bulkley model registered values greater than 100. The increase in the emulsifier content was accompanied by an increase in the consistency parameter. Although the formulation F_2_ demonstrated a higher K value, indicating increased viscosity and thickness, no significant difference was found when compared to formulation F_1_ (*p* = 0.1). The yield stress values provided by the Herschel–Bulkley model are negative (Table 5), results that we anticipated to some extent, as several other studies reported that this method often leads to negative yield stress values, which lack physical significance [40,41]. Therefore, a more robust and reproducible method such as the oscillatory stress sweep test [25] was performed to measure the yield stress of the creams.

#### 3.2.2. Dynamic Oscillatory Tests

**Figure 2:**
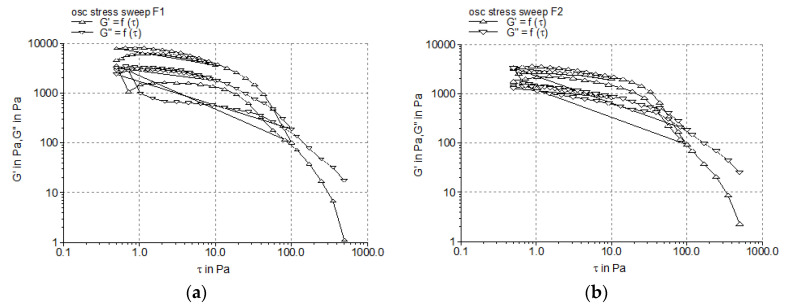
Profiles of the viscoelastic moduli G’ and G″ versus the shear stress obtained for the cream formulations: (**a**) formulation F_1_; and (**b**) formulation F_2_.

Using the oscillation amplitude sweep test, the linear viscoelastic region (LVR) of the sample can be identified. The LVR is defined as the interval of shear stress in which an oscillatory test can be carried out without destructuring the cream. The results of the oscillatory stress amplitude sweep test are shown in Figure 2, and in Table 6, the “plateau” values of the two dynamic moduli are listed.

Figure 2 exhibits the linear responses of the Montanov™-68-based creams to low shear stress values, namely approximately 0.8–2 Pa. In the LVRs of the studied creams, the profiles of the two moduli were parallel (without crossover), and the G’ values were higher than the G” values, indicating the elastic behavior of the systems. The increase of the emulsifier concentration from 5% to 7% resulted in two-fold higher G’ values (Table 6), revealing the higher stability of the internal structure of the creams containing 7% emulsifier.

**Figure 3:**
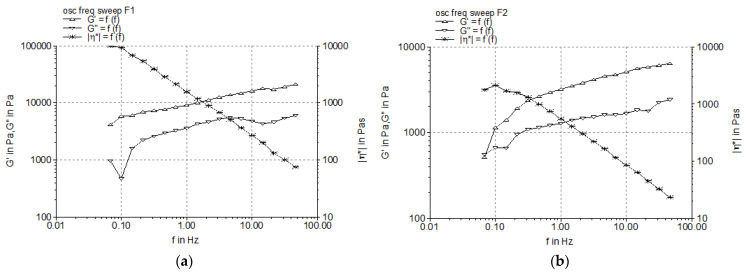
Profiles of the elastic (G’) and viscous (G″) moduli as a function of the oscillating frequency for the cream formulations F_1_ (**a**) and F_2_ (**b**).

The profiles of the elastic modulus G’ and viscous modulus G″ produced by the experimental creams when subjected to the frequency sweep test are presented in Figure 3. The results demonstrated that the G’ and G″ values increased over the frequency range (0.05–50 Hz), and the G’ was higher than the G″, revealing the larger contribution of the elastic character versus that of the viscous liquid one, and consequently, the structural strength of the complex internal network. It can be observed that by increasing the concentration of the Montanov™ 68 emulsifier, the values of both parameters increased (Figure 3).

**Figure 4:**
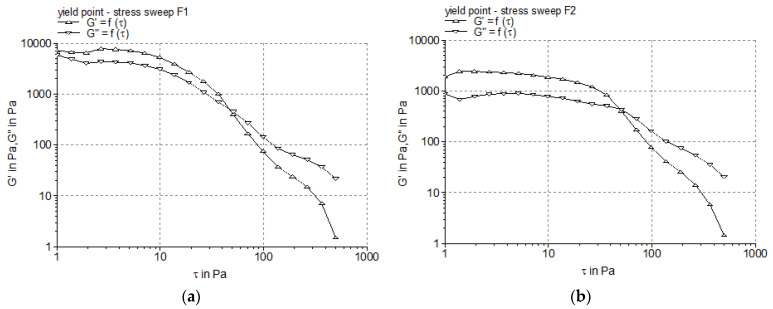
Profiles of the G’ and G″ moduli as a function of the shear stress at 1 Hz of the cream formulations F_1_ (**a**) and F_2_ (**b**).

To determine the yield stress of the tested APG-based creams containing BEO, the oscillatory stress sweep test carried out in the LVR was used. The results of this test are illustrated in Figure 4 as the profiles of the viscoelastic modules G’ and G″ as a function of the shear stress, and the obtained yield stress values are listed in Table 6.

The measured yield stress value for the F_2_ formulation was approximately two times higher in comparison to that of the F_1_ formulation (Table 6), which can be attributed to the increase in the emulsifier concentration from 5% to 7%. These results could be explained by the differences in the shear stress strength of the microstructure of the liquid crystal phases and the lamellar networks developed by the emulsifiers, which is influenced not only by the composition of the complex emulsifier but also by that of the essential oil. The results indicate that sample F_1_, exhibiting a lower yield stress, is expected to spread more easily on the skin compared to F_2_. Formulations with lower yield stress values break down more easily, which means less external force (shear stress) is necessary to deform the system and initiate its flow, which correlates with a more easy spreadability.

### 3.3. Texture Analysis

**Figure 5:**
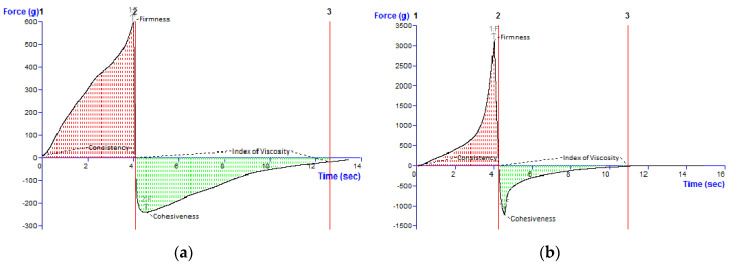
Typical force–time plots and textural parameters obtained by the back extrusion test for experimental cream formulations F_1_ (**a**) and F_2_ (**b**). (Red area refers to the total work required for compression, corresponding to the consistency parameter; Green area refers to the force required to withdraw the probe from the sample to the surface, corresponding to the viscosity index. The red lines indicate the beginning and the end of the area used to calculate the viscosity index.)

The results of the back extrusion test were used to construct the typical force vs. time profiles upon which the specific textural parameters were calculated: firmness and consistency (from the probe’s downward movement), and cohesiveness and index of viscosity (from the probe’s upward movement). The firmness and consistency quantify the ease of spreading the sample on the skin, with low parameter values indicating a more spreadable product [42,43]. The cohesiveness relates with the structuring ability of the sample after application, and the index of viscosity quantifies the adhesiveness or stickiness of the sample [44]. In terms of the typical sensory attributes that can be evaluated in the “pick-up” phase described in the Quantitative Descriptive Analysis (QDA) technique developed by Tragon Corporation [45], the cohesiveness defines “the amount of sample strings rather than breaks when fingers are separated” and the stickiness expresses the “force required to separate fingertips”. The more negative the values of the cohesiveness and index of viscosity are, the stickier the sample is. Figure 5 illustrates the back extrusion curves of the M68-based creams.

For the analyzed cream formulations based on the M68 emulsifier and containing BEO, the increase of only 2% (from 5 to 7%) in the emulsifier’s concentration produced an approx. five-fold increase in firmness, which indicates the more compact structure of the F_2_ sample. The two-fold increase in the consistency observed for sample F_2_ compared to that of F_1_ suggests greater internal resistance to deformation, which is consistent with the higher emulsifier content (7% vs. 5%) and the increased K parameter (Table 7).

In terms of the cohesiveness and index of viscosity, a similar ranking order of the experimental creams can be established, with sample F_2_ showing considerably more negative values compared to sample F_1_.

**Figure 6:**
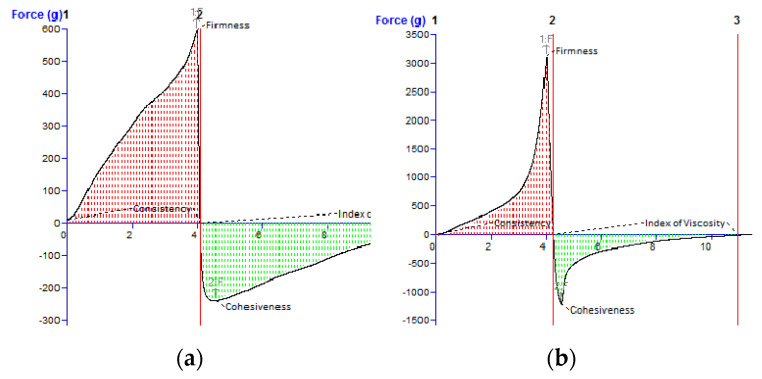
Typical force–time profiles and textural parameters obtained by the spreadability test for experimental cream formulations F_1_ (**a**) and F_2_ (**b**). (Red area refers to the shear work required for spreading, corresponding to the consistency/cohesiveness parameter; Green area refers to the force required to withdraw the probe, corresponding to the adhesiveness and viscosity index. The red lines indicate the beginning and the end of the area used to calculate the viscosity index.)

In the spreadability test performed by the texture analyzer, two textural parameters were measured: the maximum force value and the work of shear corresponding to the area under the curve in the positive region (Table 8). The specific spreadability profiles of the M68-based creams are shown in Figure 6.

According to Table 8, the values of the firmness and work of shear measured for the F_2_ formulation were approx. two times higher than those measured for the F_1_ formulation. This difference can be attributed to the increase in the emulsifier concentration from 5% to 7%, which significantly affects the lamellar networks formed by the emulsifier by producing a more compact lamellar structure, which results in the firmer structure of the cream.

### 3.4. Consistency Tests

To assess the hardness, considered a component of the structural strength (commonly named consistency) of semisolid products, the penetrometric method was used.

The penetration values for the experimental creams were similar (167 ± 1.73 mm and 151 ± 4.00 mm), indicating only a slight increase in hardness in the formulation containing the 7% emulsifier.

**Figure 7:**
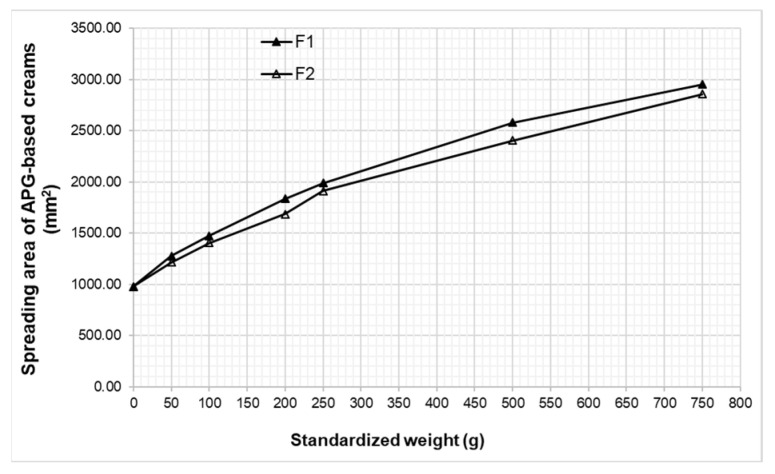
Spreadability profiles of the experimental creams obtained by the extensometric test.

The extensometric test evaluating the spreadability of the topical semisolid preparations completes the other rheological and textural tests. The variation of the cream’s spreading area with the applied weight for the experimental cream formulations is illustrated in Figure 7.

The results of the extensometric test correlate with those acquired by the penetrometric method. The extensometric profiles of the experimental creams were almost superimposable, confirming the results of the penetrometric test (the low values of the degree of penetration and similar consistency).

### 3.5. Sensory Evaluation of Cream Formulations

The results of the evaluation of the sensory characteristics of the creams based on the questionnaire filled in by the assessors were statistically analyzed. In Table 9 are presented the values of the calculated statistical parameters, including the Chi-square values (χ2), *p*-values from Friedman’s test, and rank sums with multiple comparisons using Fisher’s LSD test, for each attribute where significant differences were observed. Higher rank sums indicate a stronger perception of each sensory characteristic.

**Figure 8:**
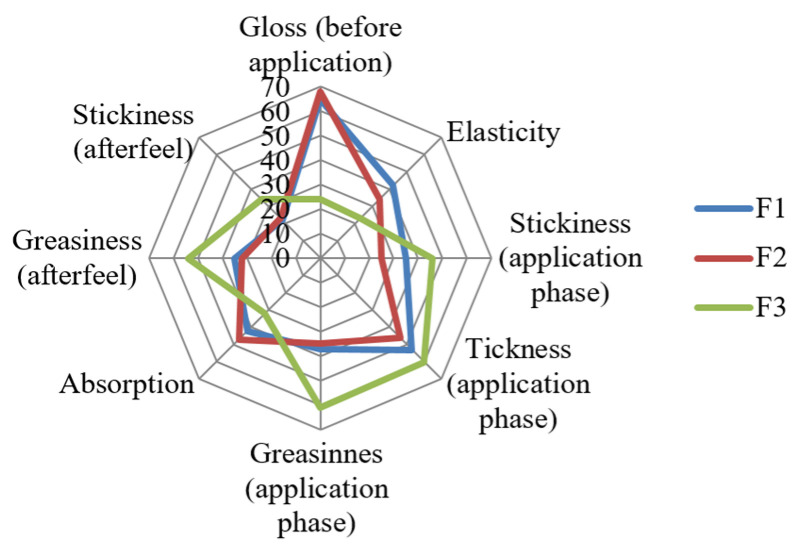
Radar chart of the rank sums of cream formulations F_1_, F_2_, and F_3_ for each sensory attribute where a significant difference was confirmed using Friedman’s test.

The rank sums of the cream samples F_1_, F_2_, and F_3_ for each of the sensory attributes where a significant difference was confirmed by Friedman’s test are shown in Figure 8.

In the phase of analysis prior to application to the skin, the samples significantly differed in gloss and elasticity. The APG-based samples were shinier and more elastic compared to sample F_3_, which was formulated with the reference base, stabilized with a synthetic non-ionic surfactant. The samples did not differ in terms of the spreadability, with the sensory analysis showing a high degree of spreadability for all the samples. Sample F_2_ (7% Montanov™ 68 emulsifier) was the least greasy and sticky during the application phase, while sample F_3_ showed the thickest structure. The sample with the highest absorption rate was identified as sample F_2_ (7% Montanov™ 68 emulsifier). In the afterfeel application to the skin, the samples significantly differed in terms of the greasiness and stickiness. Samples F_1_ and F_2_ (APG-based formulations) were significantly less greasy and sticky compared to sample F_3_, with no significant difference between samples F_1_ and F_2_. Most participants noted that all the samples left a moderate residual film on the skin. The APG-based formulations performed significantly better than the formulation with the synthetic surfactant in the following sensory attributes: absorption, stickiness during and after application, and greasiness during and after application.

## 4. Discussion

The results of the visual examination of the BEO-containing experimental creams revealed suitable homogeneity and appearance, typical of a semisolid preparation, regardless of the formulation variable (APG emulsifier concentration). However, the emulsifier content influenced the cream’s consistency.

The pH values for the tested creams remained within the recommended limits for topical preparations according to the ICH guidance (4.5–7.0) [46].

Measuring the electrical conductivity over time is considered a highly sensitive method for detecting physical changes within emulsions, where the greatest increase in conductivity over time indicates the instability of the emulsions [47]. Low values for the electrical conductivity, similar to that measured in our study, have been previously reported for APG-based emulsions and are indicative of multiphase emulsion systems [48]. A decline in the electrical conductivity of APG-based emulsions over time was explained in the literature as a consequence of the gradual binding of free water to the interlamellar spaces. Our results are consistent with the literature data on the electrical conductivity of APG-based emulsions [14,15].

The differences in terms of the viscosity and thixotropy values between the M68-stabilized creams can be attributed mainly to the resistance to flow of the cream’s internal structure, containing lamellar liquid crystal phases, formed by the mixture of alkyl polyglucoside surfactant and cetearyl alcohol as a co-surfactant. Previously published studies demonstrated that the surfactant–co-surfactant(s) mixtures’ ability to promote the formation of liquid crystals and lamellar networks varies with the number of co-surfactants in the mixture (one or more) and the co-surfactant’s chain length [49]. Moreover, in our case, the effect of the contained essential oil on the lamellar liquid structure of the creams must be taken into consideration. Various studies revealed that the rheological properties, including the viscosity, thixotropy and flow behavior, of alkyl-polyglucoside-based creams is related to their qualitative and quantitative composition, including the nature and concentration of the emulsifier, the components of the oily and aqueous phases, and the phase volume ratio [14,50].

Oscillatory shear tests examine the semisolid material under quiescent equilibrium conditions without disturbing its underlying structures, while steady shear tests can deform to a greater extent the material, thus frequently leading to false results. Therefore, it is considered that oscillatory shear tests provide more accurate information regarding the system’s rheological properties—the microstructure relationship. In the LVR, determined by the oscillation amplitude sweep test, the shear stress is directly proportional to the deformation, and the parameters describing the viscoelasticity (storage modulus G’ and loss modulus G″) remain constant (plateau value); consequently, the profiles of the viscoelastic moduli G’ and G″ versus the shear stress are registered. The limit value of the LVR region is indicated in the diagram by the rheometer software as a vertical line. The length of the LVR region indicates the cream firmness, namely, a longer LVR is correlated with a firmer (structured) cream [51,52]. The frequency sweep test provides adequate information about the microstructure of a semisolid material by evaluating its viscoelastic behavior in relation with the values of the elastic modulus G’ and viscous modulus G″. The elasticity/rigidity (structural strength) of the internal three-dimensional network is reflected by the storage modulus G’ (i.e., a higher G’ value indicates higher rigidity and structural stability), while the flexibility (viscous behavior) is indicated by the viscous modulus G″. The literature data about the rheological behavior of Montanov-68-based creams under frequency testing conditions indicate elastic solid behavior with a higher value of G’ compared to G″ at all frequencies [53,54]. Moreover, Figure 3 shows the linear decrease in the complex viscosity (η*) of both experimental creams with the increase in the oscillation frequency, which confirms the pseudoplastic (shear-thinning) behavior previously demonstrated by the steady shear test.

The yield stress is a rheological parameter with great importance in terms of the formulation stage of a pharmaceutical semisolid product, providing information about the performance, processability, and long-term stability of the product, and also about the patient acceptability. For example, the yield stress can indicate the minimum force required for the semisolid extrusion from the container, thus influencing the selection of the container; it is directly related with the behavior of the semisolid when applied onto the skin (the product should keep its initial state for a while without dripping from the skin and spread easily on the skin when fingers come into contact with its surface). In general, lower yield stress correlates with an easier spread of the product on the skin [55]. Among the methods currently used to measure the yield stress, the oscillatory stress sweep test carried out in the LVR is preferred due to its robustness and the reduced sensitivity of the sample to the “wall slip” phenomenon [25,56,57,58]. It is to be noted that the yield stress values (11.97 ± 0.26 Pa and 21.25 ± 1.14 Pa) obtained in the present study for the F_1_ and F_2_ cream formulations, respectively, were two to three times higher than those reported for other Montanov^®^-68-stabilized cream systems, for example 5.72 ± 0.86–7.45 ± 1.02 Pa [14], but this difference could be attributed mainly to the higher concentration of the consistency wax (6% cetyl palmitate versus 1% cetostearyl alcohol).

To improve the patient acceptability of topical semisolid preparations, several mechanical properties should be considered, including the extrudability from a container, spreadability on the skin, adhesiveness, and consistency [59,60]. These mechanical properties can be adequately evaluated by texture analysis, as they are quantitatively expressed as the value of external force applied to the semisolid material [61,62]. Considering that the textural properties of dermatological semisolid preparations depend on their composition, texture analysis is very useful in the formulation step of this type of preparation [63]. Consequently, to determine several important parameters (firmness, consistency, cohesiveness, and index of viscosity) related to these textural properties, the back extrusion test and spreadability test were performed in the present work. Despite the considerably higher firmness and consistency values observed for formulation F_2_ in comparison to F_1_, the difference did not reach statistical significance (*p* > 0.05), which may be attributed to the limited number of replicates and the inherent variability of texture profile measurements. The results of the back extrusion test suggest the lower spreadability of formulation F_2_; however, this was not corroborated by the sensory analysis, in which participants did not perceive any noticeable difference in spreadability between the two cream formulations.

Another important characteristic of topical semisolid formulations is their spreadability, which is defined as the ease with which the product can be applied to the desired surface (e.g., skin or mucosa). The results of the spreadability test carried out using a texture analyzer were only partially in accordance with the results of the back extrusion test. This discrepancy between the results of the back extrusion and spreadability tests can be explained by the different measurement principles, where the back extrusion test focuses on the internal structure of the sample and its resistance to compression and reverse flow, while the spreadability test is more related to the product’s behavior during application to the skin. Sample F_2_ may indeed be mechanically firmer, but the subjective sensation during spreading was not pronounced enough for the participants to perceive it as different compared to F_1_ (there was no significant difference in spreadability in the sensory evaluation).

The consistency of the studied creams was evaluated via two well-established methods: the penetrometric method and the extensometric method, measuring the hardness and the spreadability, respectively, of the sample. The penetrometric data revealed that the concentration of the Montanov emulsifier barely influenced the hardness and structural strength of the creams, with other factors such as the composition of the complex emulsifier and of the essential oil being much more important. The ratio of cetearyl alcohol to cetearyl glucoside in the complex emulsifier Montanov™ 68 may have played a more critical role in determining the textural characteristics of the creams. Cetearyl alcohol contributes to the formation of a structured, lamellar gel network, which can increase the firmness and consistency, whereas cetearyl glucoside mainly functions as a non-ionic surfactant, promoting emulsion stability without substantially increasing viscosity [64,65]. Additionally, the presence and type of the essential oil may have influenced the internal structure of the formulation by altering the polarity and fluidity of the oil phase [66]. The spreadability is considered another component of the consistency of topical semisolids, and it is usually evaluated because it indicates the ease of application of these products on the skin surface.

According to ISO 13299:2016 [67], sensory analysis is a subjective assessment that involves volunteers who, after applying the product to the skin, provide their impressions of the product’s properties [67]. When applied, semisolid formulations should spread smoothly without leaving a greasy or sticky sensation on the skin [68].

Previously published studies formulated creams containing BEO using synthetic emulsifiers (e.g., polysorbates, polyethylene glycols) [13,69]. Significant differences have been observed in the physicochemical and rheological properties of O/W creams formulated with synthetic nonionic emulsifiers compared to those containing alkyl polyglucosides. Traditional emulsifiers such as polysorbates and PEG derivatives can effectively stabilize emulsions; however, their stability often decreases over time, particularly under variable storage temperatures, leading to reduced viscosity and breakdown of the emulsion structure [70]. In comparison, APG-based emulsifiers have demonstrated superior long-term stability, primarily due to their ability to form lamellar or liquid crystalline structures, which contribute to enhanced viscoelastic behavior and moisture retention [31]. Additionally, formulations using APGs are generally considered milder and biocompatible, making them especially suitable for sensitive skin and “green” formulations [15,71]. These findings support the growing preference for APGs in modern dermatological and cosmetic form for biocompatibility, safety, and sustainability.

## 5. Conclusions

To the best of our knowledge, this research is the first to report formulations of an alkyl-polyglucosides-based cream containing basil essential oil, specifically using Montanov™ 68 as the emulsifier, and their characterization in terms of the physicochemical, mechanical, and sensory properties. Montanov™ 68 was found to be successful in emulsifying BEO to develop physically stable creams, with dermatologically accepted pH values, exhibiting non-Newtonian behavior characterized by pseudoplasticity and thixotropy. Increasing the emulsifier content from 5% to 7% doubled the storage modulus (G’), indicating a stronger internal structure, while the 5% formulation showed lower yield stress, suggesting easier spreadability. Higher emulsifier levels corresponded to increased firmness, consistency, cohesiveness, and viscosity. The rheological analysis suggested the superior spreadability of the formulation with 5% emulsifier; however, this finding was not reflected in the sensory analysis, possibly because the observed difference was not significant enough to be subjectively detected. The sensory analysis revealed that the APG-based creams differed mainly in elasticity and stickiness, while both outperformed the synthetic surfactant formulation in terms of the absorption, stickiness, and greasiness during and after application.

All these experimental results are encouraging for continuing the evaluation of the formulated Montanov™-68-based creams containing basil essential oils in terms of the in vitro release/permeation of active ingredient, safety in topical application, and in vitro/in vivo biological activity.

## Figures and Tables

**Table 1:** Composition of the cream formulations (*w*/*w* %).

Ingredient	Functional Category	Content (*w*/*w* %)			
		F_1_	F_2_	F_3_	Control Sample
**Oil phase**					
Caprylic/capric triglyceride	Emollient	10.00	10.00	-	-
Cetyl palmitate	Emollient	6.00	6.00	-	-
Almond oil	Emollient	5.00	5.00	-	-
Montanov™ 68	O/W emulsifier	5.00	7.00	-	-
Tocopheryl acetate	Antioxidant	0.50	0.50	-	-
**Water phase**					
Sodium sorbate	Preservative	0.50	0.50	0.50	-
Xanthan gum	Co-emulsifier	0.50	0.50	-	-
Propylene glycol	Humectant	4.00	4.00	-	-
**Active ingredient**					
Basil essential oil		1.00	1.00	1.00	1.00
**Non-ionic hydrophilic base**	**Base**				
Polysorbate 60	O/W emulsifier	-	-	-	2.00
Cetylstearyl alcohol	Co-emulsifier	-	-	-	4.00
Glycerol	Humectant	-	-	-	4.00
Vaseline	Emollient	-	-	-	10.00
Purified water	Solvent	to 100.00	to 100.00	to 100.00	to 100.00

F_1_—5% Montanov™ 68 and 1% BEO; F_2_—7% Montanov™ 68 and 1% BEO.

**Table 2:** Sensory attributes questionnaire used for the evaluation.

Before the application	Consistency	liquid/semisolid
	Gloss level	matte/pearl gloss/slightly glossy/gloss/very glossy
	Adhesion—the amount of the sample that remains on the index finger after brief contact of 2 s (scale)	1–10
	Elasticity—the degree of stretching of the sample between the thumb and index finger.	slightly elastic/elastic/very elastic
	Texture—the impression of the thickness of the sample when rubbed between the thumb and index finger (scale)	1–10
Application phase	Spreadability—the degree of spreadability and melting of the sample when rubbed on the skin of the palm in circular motions 2 times (scale)	1–10
	Stickiness—the force required to separate the finger from the skin	not sticky/slightly sticky/sticky/very sticky
	Thickness—the degree of density during application	thin/slightly thick/thick/very thick
	Greasiness—the degree of grease during application	not greasy/slightly greasy/greasy/very greasy
	Gloss—the degree of gloss during application	not shiny/slightly shiny/shiny/very shiny
	Absorption—the impression of the sample absorption rate	slow/moderate/fast
	Residual film—the impression of residual film on the skin 10 min after application	no film/moderate/pronounced
After application	Greasiness—the impression of skin being greasy 10 min after application	not greasy/slightly greasy/greasy/very greasy
	Gloss—the degree of skin gloss after application	not shiny/slightly shiny/shiny/very shiny
	Stickiness—the impression of a sticky feeling on the skin 10 min after application	not sticky/slightly sticky/sticky/very sticky

**Table 3:** pH and electrical conductivity (µS/cm) values of the cream samples at 7, 30, and 90 days after production, as well as after the accelerated aging stability test.

**Formulation Code**	**After 7 Days**	**After 30 Days**	**After 90 Days**	**After the Accelerated Aging Test**
**pH**				
F_1_	6.83	6.80	6.60	6.61
F_2_	6.81	6.78	6.57	6.55
**Electrical conductivity**				
F_1_	12.33	11.56	11.10	10.96
F_2_	13.45	12.60	10.72	10.70

**Table 4:** Apparent viscosity and thixotropy index values of the experimental creams.

Formulation Code	Apparent Viscosity (Pa.s)	Thixotropy Index (%)
F_1_	2.182 ± 0.064	17.961 ± 2.504
F_2_	2.325 ± 0.071	22.176 ± 3.016

**Table 5:** Rheological parameters of the Ostwald de Waele and Herschel–Bulkley models.

Formulation Code	Model Correlation Coefficient (R^2^)		Parameters of Ostwald de Waele Model		Parameters of Herschel–Bulkley Model		
	Ostwald de Waele	Herschel–Bulkley	K	n	K	n	τ_0_ (Pa)
F_1_	0.9330 ± 0.004	0.9363 ± 0.003	63.83 ± 2.09	0.2675 ± 0.012	111.5 ± 1.34	0.1948 ± 0.011	−55.78 ± 0.82
F_2_	0.8770 ± 0.007	0.884 ± 0.005	79.83 ± 1.77	0.2346 ± 0.008	175.3 ± 1.47	0.1433 ± 0.005	−105.20 ± 1.17

**Table 6:** The results of the amplitude sweep test for the cream formulations and the yield stress values.

Formulation Code	Elastic Modulus, G’ (Pa)	Viscous Modulus, G” (Pa)	Yield Stress, τ_0_ (Pa)
F_1_	5334.10 ± 105.23	2591.20 ± 78.43	11.97 ± 0.26
F_2_	2557.95 ± 61.47	1211.31 ± 32.86	21.25 ± 1.14

**Table 7:** Textural parameter values of the experimental creams determined in the back extrusion test (values expressed as mean ± standard deviation, *n* = 3).

Formulation Code	Firmness (g)	Consistency (g·sec)	Cohesiveness (g)	Index of Viscosity (g·sec)
F_1_	596.39 ± 10.71	1187.52 ± 15.48	−242.75 ± 6.81	−967 ± 9.11
F_2_	3115.02 ± 16.29	2823.35 ± 14.87	−1219.82 ± 12.44	−1601.72 ± 13.25

**Table 8:** Textural parameter values of the experimental creams determined in the spreadability test (values expressed as mean ± standard deviation, *n* = 3).

Formulation Code	Firmness (g)	Work of Shear (g·sec)
F_1_	1216.47 ± 12.30	1090.03 ± 32.55
F_2_	2153.36 ± 15.37	2627.81 ± 20.16

**Table 9:** Sensory evaluation of the APG-based cream formulations.

Attribute	χ2	*p*	Rank Sum		
			F_1_	F_2_	F_3_
**Before the application** Gloss Elasticity					
	34.939	<0.001 **	65 ^a^	68 ^a^	24 ^b^
	20.548	<0.001 **	42 ^a^	34 ^b^	23 ^c^
**During the application** Stickiness Thickness Greasiness Absorption					
	16.095 7.600 32.281 14.157	<0.001 ** 0.022 * <0.001 ** 0.001 **	36 ^a^ 53 37 ^a^ 42 ^a^	25 ^b^ 46 ^a^ 35 ^a^ 47 ^a^	46 ^c^ 60 ^b^ 61 ^b^ 32 ^b^
**Afterfeel phase** Greasiness Stickiness					
	21.815	<0.001 **	35 ^a^	32 ^a^	54 ^b^
	16.267	<0.001 **	22 ^a^	23 ^a^	34 ^b^

Different letters (a, b, c) in superscript in the same row significantly differ from each other at * *p* < 0.05 and ** *p* < 0.01. F_1_—M68 5% + BEO 1%, F_2_ M68 7% + BEO 1%, F_3_—reference non-ionic hydrophile base + BEO 1%.

## Data Availability

The authors confirm that the data supporting the findings of this study are available within the article.

## Abbreviations

The following abbreviations are used in this manuscript:

BEO	Basil essential oil
APG	Alkyl polyglucoside
O/W	Oil-in-water
FDA	Food and Drug Administration
LVR	Linear viscoelasticity region
ICH	International Council for Harmonisation of Technical Requirements for Pharmaceuticals for Human Use

## References

[1] Zagoto M., Cardia G.F.E., da Rocha E.M.T., Mourão K.S.M., Janeiro V., Cuman R.K.N., Pinto A.A., Contiero R.L., de Freitas P.S.L. (2021). Biological Activities of Basil Essential Oil: A Review of the Current Evidence. Res. Soc. Dev..

[2] World Health Organization (2023). Integrating Traditional Medicine in Health Care.

[3] Valiakos E., Marselos M., Sakellaridis N., Constantinidis T., Skaltsa H. (2015). Ethnopharmacological Approach to the Herbal Medicines of the “Antidotes” in Nikolaos Myrepsos׳ Dynameron. J. Ethnopharmacol..

[4] Dharsono H.D.A., Putri S.A., Kurnia D., Dudi D., Satari M.H. (2022). Ocimum Species: A Review on Chemical Constituents and Antibacterial Activity. Molecules.

[5] Azizah N.S., Irawan B., Kusmoro J., Safriansyah W., Farabi K., Oktavia D., Doni F., Miranti M. (2023). Sweet Basil (Ocimum Basilicum L.)-A Review of Its Botany, Phytochemistry, Pharmacological Activities, and Biotechnological Development. Plants.

[6] Sakkas H., Papadopoulou C. (2017). Antimicrobial Activity of Basil, Oregano, and Thyme Essential Oils. J. Microbiol. Biotechnol..

[7] Zhakipbekov K., Turgumbayeva A., Akhelova S., Bekmuratova K., Blinova O., Utegenova G., Shertaeva K., Sadykov N., Tastambek K., Saginbazarova A. (2024). Antimicrobial and Other Pharmacological Properties of Ocimum Basilicum, Lamiaceae. Molecules.

[8] Amor G., Sabbah M., Caputo L., Idbella M., De Feo V., Porta R., Fechtali T., Mauriello G. (2021). Basil Essential Oil: Composition, Antimicrobial Properties, and Microencapsulation to Produce Active Chitosan Films for Food Packaging. Foods.

[9] Rodrigues L.B., Oliveira Brito Pereira Bezerra Martins A., Cesário F.R.A.S., Ferreira E Castro F., de Albuquerque T.R., Martins Fernandes M.N., Fernandes da Silva B.A., Quintans Júnior L.J., da Costa J.G.M., Melo Coutinho H.D. (2016). Anti-Inflammatory and Antiedematogenic Activity of the Ocimum Basilicum Essential Oil and Its Main Compound Estragole: In Vivo Mouse Models. Chem. Biol. Interact..

[10] Robu A., Kaya M.G.A., Antoniac A., Kaya D.A., Coman A.E., Marin M.-M., Ciocoiu R., Constantinescu R.R., Antoniac I. (2025). The Influence of Basil and Cinnamon Essential Oils on Bioactive Sponge Composites of Collagen Reinforced with Hydroxyapatite. Materials.

[11] Sestili P., Ismail T., Calcabrini C., Guescini M., Catanzaro E., Turrini E., Layla A., Akhtar S., Fimognari C. (2018). The Potential Effects of Ocimum Basilicum on Health: A Review of Pharmacological and Toxicological Studies. Expert Opin. Drug Metab. Toxicol..

[12] Stanojević L., Stanojević J., Savić V., Cvetkovic D., Kolarevic A., Marjanović-Balaban Ž., Nikolic L. (2019). Peppermint and Basil Essential Oils: Chemical Composition, in Vitro Antioxidant Activity and in Vivo Estimation of Skin Irritation. J. Essent. Oil Bear. Plants.

[13] Irianto I., Ismiyati, Witaningrum E., Ayuningtyas E., Ulfah M., Purwanto P. (2023). Antibacterial Activity of Cream, Ointment, and Emulgel of Ocimum Basilicum L. Essential Oil against Propionibacterium Acnes. Maj. Obat Tradis..

[14] Jaksic I., Lukic M., Malenovic A., Reichl S., Hoffmann C., Müller-Goymann C., Daniels R., Savic S. (2012). Compounding of a Topical Drug with Prospective Natural Surfactant-Stabilized Pharmaceutical Bases: Physicochemical and in Vitro/in Vivo Characterization—A Ketoprofen Case Study. Eur. J. Pharm. Biopharm. Off. J. Arbeitsgemeinschaft Pharm. Verfahrenstechnik EV.

[15] Ilic D., Cvetkovic M., Tasic-Kostov M. (2021). Emulsions with Alkyl Polyglucosides as Carriers for Off-Label Topical Spironolactone—Safety and Stability Evaluation. Pharm. Dev. Technol..

[16] MONTANOV 68 MB. https://www.seppic.com/en-US/product/montanov-68-mb/01tD0000005kfP8IAI.

[17] Jarupinthusophon S., Preechataninrat P., Anurukvorakun O. (2022). Development of Liquid Crystal Cream Containing Germinated Brown Rice. Appl. Sci..

[18] How Lamellar Liquid Crystal Emulsion Can Strengthen Skin Barrier?. https://www.seppic.com/en-US/article/how-lamellar-liquid-crystal-emulsion-can-strengthen-skin-barrier#:~:text=MONTANOV%E2%84%A2%20can%20be%20added,formation%20of%20lamellar%20liquid%20crystals.

[19] Sladmin Europäisches und Deutsches Arzneibuch. https://www.deutsche-apotheker-zeitung.de/daz-az/2006/daz-3-2006/uid-15294.

[20] Aulton M.E., Taylor K.M. (2017). Aulton’s Pharmaceutics: The Design and Manufacture of Medicines.

[21] Directorate for the Quality of Medicines, Healthcare of the Council of Europe (2022). Potentiometric Determination of pH. European Pharmacopeia.

[22] Directorate for the Quality of Medicines, Healthcare of the Council of Europe (2022). Conductivity 2.2.38. European Pharmacopoeia.

[23] Ghica M.V., Hîrjău M., Lupuleasa D., Dinu-Pîrvu C.-E. (2016). Flow and Thixotropic Parameters for Rheological Characterization of Hydrogels. Molecules.

[24] Calienni M.N., Martínez L.M., Izquierdo M.C., Alonso S.d.V., Montanari J. (2023). Rheological and Viscoelastic Analysis of Hybrid Formulations for Topical Application. Pharmaceutics.

[25] Dinkgreve M., Paredes J., Denn M.M., Bonn D. (2016). On Different Ways of Measuring “the” Yield Stress. J. Non-Newton. Fluid Mech..

[26] Perrot A., Mélinge Y., Estellé P., Rangeard D., Lanos C. (2019). The Back Extrusion Test as a Technique for Determining the Rheological and Tribological Behaviour of Yield Stress Fluids at Low Shear Rates. Appl. Rheol..

[27] Directorate for the Quality of Medicines, Healthcare of the Council of Europe (2022). Measurement of Consistency by Penetrometry. European Pharmacopeia.

[28] Parente M.E., Ochoa Andrade A., Ares G., Russo F., Jiménez-Kairuz Á. (2015). Bioadhesive Hydrogels for Cosmetic Applications. Int. J. Cosmet. Sci..

[29] Standard Guide for Two Sensory Descriptive Analysis Approaches for Skin Creams and Lotions. https://store.astm.org/e1490-19.html.

[30] Tadić V.M., Žugić A., Martinović M., Stanković M., Maksimović S., Frank A., Nešić I. (2021). Enhanced Skin Performance of Emulgel vs. Cream as Systems for Topical Delivery of Herbal Actives (Immortelle Extract and Hemp Oil). Pharmaceutics.

[31] Lukic M., Jaksic I., Krstonosic V., Cekic N., Savic S. (2012). A Combined Approach in Characterization of an Effective w/o Hand Cream: The Influence of Emollient on Textural, Sensorial and in Vivo Skin Performance. Int. J. Cosmet. Sci..

[32] Hassan A.K. (2015). Effective Surfactants Blend Concentration Determination for O/W Emulsion Stabilization by Two Nonionic Surfactants by Simple Linear Regression. Indian. J. Pharm. Sci..

[33] Stojiljković D., Arsić I., Tasić-Kostov M. (2016). The influence of polar and non-polar emollients on the structure and skin moisturizing potential of the emulsions stabilized by mixed emulsifier. Acta Med. Median..

[34] Petrovic B., Bradic J., Petrovic A., Ivanovic D., Tabakovic M., Saric S., Jakovljevic V. (2017). Developement and Stability Evaluation of Natural Topical Formulations Containing *Pinus sibirica* Essential Oil. Exp. Appl. Biomed. Res. (EABR) Sciendo..

[35] Anton N., Gayet P., Benoit J.P., Saulnier P. (2007). Nano-emulsions and nanocapsules by the PIT method: An investigation on the role of the temperature cycling on the emulsion phase inversion. Int. J. Pharm..

[36] Allouche J., Tyrode E., Sadtler V., Choplin L., Salager J.L. (2004). Simultaneous conductivity and viscosity measurements as a technique to track emulsion inversion by the phase-inversion-temperature method. Langmuir.

[37] Larson R., Wei Y. (2019). A Review of Thixotropy and Its Rheological Modeling. J. Rheol..

[38] European Medicines Agency (2018). Draft Guideline on Quality and Equivalence of Topical Products.

[39] Block L.H., Felton L. (2012). Rheology. Remington Essentials of Pharmaceutics.

[40] Magnon E., Cayeux E. (2021). Precise Method to Estimate the Herschel-Bulkley Parameters from Pipe Rheometer Measurements. Fluids.

[41] Kelessidis V.C., Maglione R., Tsamantaki C., Aspirtakis Y. (2006). Optimal determination of rheological parameters for Herschel–Bulkley drilling fluids and impact on pressure drop, velocity profiles and penetration rates during drilling. J. Pet. Sci. Eng..

[42] Akanny E., Kohlmann C. (2024). Predicting Tactile Sensory Attributes of Personal Care Emulsions Based on Instrumental Characterizations: A Review. Int. J. Cosmet. Sci..

[43] Mazurkevičiūtė A., Matulytė I., Ivaškienė M., Žilius M. (2022). Assessment of Physical, Mechanical, Biopharmaceutical Properties of Emulgels and Bigel Containing Ciclopirox Olamine. Polymers.

[44] Huynh A., Garcia A.G., Young L.K., Szoboszlai M., Liberatore M.W., Baki G. (2021). Measurements Meet Perceptions: Rheology-Texture-Sensory Relations When Using Green, Bio-Derived Emollients in Cosmetic Emulsions. Int. J. Cosmet. Sci..

[45] Sidel J.L., Bleibaum R.N., Clara Tao K.W. (2018). Quantitative Descriptive Analysis. Descriptive Analysis in Sensory Evaluation.

[46] Abraham J., Tietje C., Brouder A. (2010). International Conference On Harmonisation Of Technical Requirements For Registration Of Pharmaceuticals For Human Use. Handbook of Transnational Economic Governance Regimes.

[47] Zhang W., Yiu Y., Lin M., Luo T., Yao C. (2008). Electrical Conductivity and Stability of O/W Emulsions. Shiyou Xuebao Shiyou JiagongActa Pet. Sin. Pet. Process. Sect..

[48] Salager J.-L., Nielloud F., Marti-Mestres G. (2000). Emulsion Properties and RelatedKnow-how to Attain Them. Pharmaceutical Emulsions and Suspensions.

[49] Ahmadi D., Mahmoudi N., Heenan R.K., Barlow D.J., Lawrence M.J. (2020). The Influence of Co-Surfactants on Lamellar Liquid Crystal Structures Formed in Creams. Pharmaceutics.

[50] Wojciechowska K., Walczak A., Rostowska E., Poleszak E. (2021). Comparison of Sensory and Rheological Properties of Green Cosmetic Creams Prepared on Different Natural, ECOCERT and BDIH Certificated Self-Emulsifying Bases. Curr. Issues Pharm. Med. Sci..

[51] Koruk H., Rajagopal S. (2024). A Comprehensive Review on the Viscoelastic Parameters Used for Engineering Materials, Including Soft Materials, and the Relationships between Different Damping Parameters. Sensors.

[52] Adejokun D.A., Dodou K. (2020). Quantitative Sensory Interpretation of Rheological Parameters of a Cream Formulation. Cosmetics.

[53] Masmoudi H., Piccerelle P., Le Dréau Y., Kister J. (2006). A Rheological Method to Evaluate the Physical Stability of Highly Viscous Pharmaceutical Oil-in-Water Emulsions. Pharm. Res..

[54] Vitek M., Medoš Ž., Lavrič Z., Jeras M., Planinšek O., Pobirk A.Z., Matjaž M.G. (2024). Highly Biocompatible Lamellar Liquid Crystals Based on Hempseed or Flaxseed Oil with Incorporated Betamethasone Dipropionate: A Bioinspired Multi-Target Dermal Drug Delivery System for Atopic Dermatitis Treatment. Int. J. Nanomed..

[55] Kwak M.-S., Ahn H.-J., Song K.-W. (2015). Rheological Investigation of Body Cream and Body Lotion in Actual Application Conditions. Korea-Aust. Rheol. J..

[56] Semancik J.R. Yield Stress Measurements Using Controlled Stress Rheometry. Rheological Techniques for Yield Stress Analysis.

[57] Calderas F., Herrera-Valencia E.E., Sanchez-Solis A., Manero O., Medina-Torres L., Renteria A., Sanchez-Olivares G. (2013). On the Yield Stress of Complex Materials. Korea-Aust. Rheol. J..

[58] Tamburic S., Sisson H., Cunningham N., Stevic M.C. (2017). Rheological and Texture Analysis Methods for Quantifying Yield Value and Level of Thixotropy. SOFW J..

[59] Oliveira R., Almeida I.F. (2023). Patient-Centric Design of Topical Dermatological Medicines. Pharmaceuticals.

[60] European Medicines Agency (2014). Guideline on Quality and Equivalence of Locally Applied, Locally Acting Cutaneous Products.

[61] Tai A., Bianchini R., Jachowicz J. (2014). Texture Analysis of Cosmetic/Pharmaceutical Raw Materials and Formulations. Int. J. Cosmet. Sci..

[62] AL-Smadi K., Ali M., Zhu J., Abdoh A., Phan K., Mohammed Y. (2025). Advances in Characterization of Transdermal and Topical Products using Texture Analyzer Systems. AAPS PharmSciTech.

[63] Siemiradzka W., Dolińska B., Ryszka F. (2020). Development and Study of Semi-Solid Preparations Containing the Model Substance Corticotropin (ACTH): Convenience Application in Neurodegenerative Diseases. Molecules.

[64] Jiménez Soriano M.M., Fresno Contreras M.J., Sellés Flores E. (2001). Development of a Cream from a Self-Emulsifying Base and Moisturizing Actives. Farm. Soc. Chim. Ital. 1989.

[65] Morávková T., Filip P. (2013). The Influence of Emulsifier on Rheological and Sensory Properties of Cosmetic Lotions. Adv. Mater. Sci. Eng..

[66] Ganguly R., Verma G., Ingle A., Kumar S., Sarma H.D., Dutta D., Dutta B., Kunwar A., Ajish K., Bhainsa K.C. (2022). Structural, Rheological and Therapeutic Properties of Pluronic F127 Hydrogel and Beeswax Based Lavender Oil Ointment Formulations. J. Mol. Liq..

[67] (2016). Sensory Analysis—Methodology—General Guidance for Establishing a Sensory Profile.

[68] Garg A., Aggarwal D., Garg S., Singla A.K. (2002). Spreading of Semisolid Formulations. Pharm. Technol. N. Am..

[69] Yadav N.P., Meher J.G., Pandey N., Luqman S., Yadav K.S., Chanda D. (2013). Enrichment, Development, and Assessment of Indian Basil Oil Based Antiseptic Cream Formulation Utilizing Hydrophilic Lipophilic Balance Approach. Biomed. Res. Int..

[70] Korhonen M., Hirvonen J., Yliruusi J. (2001). Rheological properties of creams with four different surfactant combinations—effect of storage time and conditions. Int. J. Pharm..

[71] Hanno I., Centini M., Anselmi C., Bibiani C. (2015). Green Cosmetic Surfactant from Rice: Characterization and Application. Cosmetics.

